# Disease Models & Mechanisms in 2016: a publisher's brief perspective

**DOI:** 10.1242/dmm.024620

**Published:** 2016-02-01

**Authors:** Rachel Hackett

**Affiliations:** Disease Models & Mechanisms,The Company of Biologists, Bidder Building, Station Road, Histon, Cambridge CB24 9LF, UK

## Abstract

**Summary:** DMM announces changes to its Senior Editor team and thanks its many reviewers.

“*Journals do more than publish collections of research articles and reviews; they also create community, influence standards for community behavior and ‘provoke progress’ in a particular field*” –

Vivian Siegel, Founding Editor-in-Chief at DMM.

In 2008, then Disease Models & Mechanisms (DMM) Editor-in-Chief Vivian Siegel likened launching the journal to raising a child (Siegel, 2008). Five years later, Vivian described how she was letting go of the reins, transferring editorial responsibilities to a new team of expert researchers perfectly matched to DMM's remit of publishing high-quality research that describes the use of model organisms to understand, diagnose and treat human disease (Siegel, 2013). This team of academic editors was to be led by Ross Cagan as Editor-in-Chief, with Monica Justice and George Tidmarsh as Senior Editors.

When this new editorial team was established, it was done so with the intention that the Editor-in-Chief role would rotate. So, as of 1 January 2016, Monica Justice became Editor-in-Chief, with Ross Cagan stepping into the Senior Editor role. Ross and Monica will continue to work together – in collaboration with the publishing team in Cambridge, UK and the dedicated team of Monitoring Editors (Box 1) – to build on the successes the journal has seen under Ross's leadership over the past 3 years.

**Box 1. DMM Editor team 2016**

**Editor-in-Chief**

Monica J. Justice [The Hospital for Sick Children (SickKids), Toronto, Canada]

**Senior Editor**

Ross Cagan (Icahn School of Medicine at Mount Sinai, NY, USA)

**Monitoring Editors**

Steven J. Clapcote (University of Leeds, UK)

Pamela Hoodless (BC Cancer Agency, Vancouver, Canada)

Tatsushi Igaki (Kyoto University, Japan)

Elaine Mardis (Genome Institute at Washington University School of Medicine, MO, USA)

E. Elizabeth Patton (MRC Institute of Genetics and Molecular Medicine, Edinburgh, UK)

Owen Sansom (Beatson Institute for Cancer Research, Glasgow, UK)

David Tobin (Duke University School of Medicine, Durham, NC, USA)

The new team was charged with guiding the journal to become a platform for research that bridges the gap between basic biological insights and drug discovery and development. To this end, the team has taken efforts to ensure that high standards for translational impact are enforced while implementing fair and transparent editorial practices, and continuing to engage and inform readers with a diverse ‘front section’ comprising timely reviews, interviews and educational poster articles. Their vision for the journal – roughly summarised as helping it to become the ‘go-to’ journal for research using disease models (Cagan et al., 2013) – has started to become a reality.

We have seen the number of submissions to DMM double, leading to a move to monthly rather than bimonthly publication. DMM articles were accessed over 1 million times in 2015. We have introduced special subject collections, covering both disease areas, such as cancer and metabolic disorders, and the translational impact of model systems, such as zebrafish, model systems in drug discovery and, coming up in 2016, *Drosophila* and rat. The Impact Factor and other journal- and article-level metrics are more than respectable. Importantly, we have focused on maintaining high standards for the quality of articles that we publish. The work must of course be sound, but it must also give significant new insights into the mechanisms, diagnosis and/or treatment of disease using model systems. We encourage authors to develop their studies beyond descriptive work, such as the characterisation of a model system without a demonstration of its utility, so that the work we publish will have a strong translational impact. DMM also plays a crucial role in promoting standards for reproducibility and proper reporting to ensure that the use of model organisms advances translational research in the most efficient and effective way (Siegel, 2011; Matosin et al., 2014; Justice and Dhillon, 2016).

Ross is still very much involved in the running of the journal with Monica [read their absorbing A Model for Life interviews to find out more about them (Justice, 2013; Cagan, 2013)]. DMM's team of Editors has always worked very collaboratively and this will, of course, continue. Ross will also be kept busy organising a Company of Biologists' workshop later this year – on Rethinking Cancer (http://www.biologists.com/workshops/rethinking-cancer-november-2016/). DMM will be there, sharing the highlights and commissioning timely articles from leading experts. We will also be attending the Allied GSA meeting in Florida in July 2016 (http://www.genetics2016.org/), in line with our goal to support collaboration across the different disciplines and model communities.

The need for the broad scientific community of disease researchers and clinicians to communicate effectively remains. DMM has and will continue to strive to achieve this and improve the translation of basic research to the clinic. None of this would be possible without the many researchers who have submitted to and published in DMM, and the journal Editors, Editorial Board members and staff. Their names are public knowledge, but those of our reviewers are not. Many journals are undertaking experiments to improve or refine the peer review process, as well as holding conversations about how to best reward and recognise the vital work done by so many. We take a small step in this direction by publishing the names of all those who completed a review for DMM during 2015, with our sincere thanks for their expertise and for managing to fit one more task into their already busy schedules (Table S1).
